# Low-dose tributyltin exposure induces an oxidative stress-triggered JNK-related pancreatic β-cell apoptosis and a reversible hypoinsulinemic hyperglycemia in mice

**DOI:** 10.1038/s41598-018-24076-w

**Published:** 2018-04-10

**Authors:** Chun-Fa Huang, Ching-Yao Yang, Jing-Ren Tsai, Cheng-Tien Wu, Shing-Hwa Liu, Kuo-Cheng Lan

**Affiliations:** 1 School of Chinese Medicine, College of Chinese Medicine, China Medical University, Taichung, Taiwan; 2 Department of Nursing, College of Medical and Health Science, Asia University, Taichung, Taiwan; 3 Department of Surgery, College of Medicine and Hospital, National Taiwan University, Taipei, Taiwan; 4 Institute of Toxicology, College of Medicine, National Taiwan University, Taipei, Taiwan; 5 Department of Pediatrics, College of Medicine and Hospital, National Taiwan University, Taipei, Taiwan; 6 Department of Medical Research, China Medical University Hospital, China Medical University, Taichung, Taiwan; 7 Department of Emergency Medicine, Tri-Service General Hospital, National Defense Medical Center, Taipei, Taiwan

## Abstract

Tributyltin (TBT), an endocrine disrupting chemical, can be found in food (particular in fish and seafood) and drinking water by contamination. Here, we elucidated the effects and possible mechanisms of low-dose TBT on the growth and function of pancreatic β-cells and glucose metabolism in mice. Submicromolar-concentration of TBT significantly induced β-cell cytotoxicity and apoptosis, which were accompanied by poly (ADP-ribose) polymerase cleavage and mitogen-activated protein kinases-JNK and ERK1/2 phosphorylation. TBT could also suppress the glucose-stimulated insulin secretion in β-cells and isolated mouse islets. TBT increased reactive oxygen species production. TBT-induced β-cell cytotoxicity and apoptosis were significantly prevented by antioxidant N-acetylcysteine (NAC) and JNK inhibitor SP600125, but not ERK1/2 inhibitor PD98059 and p38 inhibitor SB203580. Both NAC and SP600125 inhibited JNK phosphorylation and reduced cell viability in TBT-treated β-cells. Four-week exposure of TBT (0.25 mg/kg) to mice revealed the decreased plasma insulin, increased blood glucose and plasma malondialdehyde, suppressed islet insulin secretion, and increased islet caspase-3 activity, which could be reversed by NAC treatment. After removing the TBT exposure for 2 weeks, the TBT-induced glucose metabolism alteration was significantly reversed. These results suggest that low-dose TBT can induce β-cell apoptosis and interfere with glucose homeostasis via an oxidative stress-related pathway.

## Introduction

Endocrine disrupting chemicals (EDCs) are chemical compounds that mimic or interfere with the synthesis, secretion, transport, function, or metabolism of natural hormones, causing a wide range and deleterious effects in physiological systems including reproductive, neurological, cardiovascular, metabolic and immune systems^1,2^. The relationship of environmental chemicals as characterized by EDCs with obesity, diabetes mellitus, and metabolic syndrome has been comprehensively evaluated from the data of epidemiological and experimental studies^3^. The increased presence of EDCs in the life environment of humans has been reported playing an important role in the disruption of pancreatic β-cells function and the development of diabetes-related diseases^4,5^.

Organotin compounds are widely used as the plastic stabilizers/catalysts in industry and the biocides in agriculture. The environmental pollution of tributyltin (TBT) has been tremendously pervaded to cause the dramatic exposure and health risk in human that the extensive use of TBT leading to mammal exposure to occur through the bioaccumulation and biomagnification of contaminated dietary sources (seafood and drinking water)^6,7^. The application of TBT such as the marine antifouling paints has been prohibited because of the highly stable and resistant to natural degradation in water^7,8^. TBT is known as potential human EDCs. On the basis of the no observed effect level of 0.5 mg/kg bw from the results of short-term toxicity tests using 100 as a safety factor, the tolerable daily intake for TBT oxide has been estimated to be 5 μg/kg bw per day^9^.

TBT has been concerned to possess the *in vitro* and *in vivo* deleterious effects like as neurological, immunological, and hepatic toxicities^10–12^. Several studies have also shown that exposure of experimental animals to TBT can trigger insulin dysregulation and disturb glucose homeostasis^13–16^. The effect of TBT exposure on the alteration of insulin secretion in mammalian may contribute to an environment risk factor in the development of diabetes. However, the detailed toxicological effects and mechanisms underlying TBT-triggered pancreatic islet β-cell injury remain for further investigation.

Pancreatic islet β-cell cells are vulnerable to oxidative stress, which may induce β-cell apoptosis and β-cell mass reduction, resulting in the dysfunction of insulin secretion and the pathogenesis of diabetes^17^. Chemicals, which induce overproduction of reactive oxygen species (ROS), are known to aggravate the diabetic situation and act as a predisposing factor for diabetes. Several *in vitro* and *in vivo* studies have shown that oxidative damage is a major insult of TBT toxicity^12,18,19^. However, the key role of ROS in TBT-induced islet β-cell injury remains unclear. Therefore, we aimed to examine the effects of TBT on islet β-cell dysfunction and apoptosis *in vitro* and *in vivo* and investigate the involvement of ROS-mediated molecular signals in these TBT-induced effects.

## Results

### TBT alters growth and function and induces apoptosis in RIN-m5F cells

**Figure 1:**
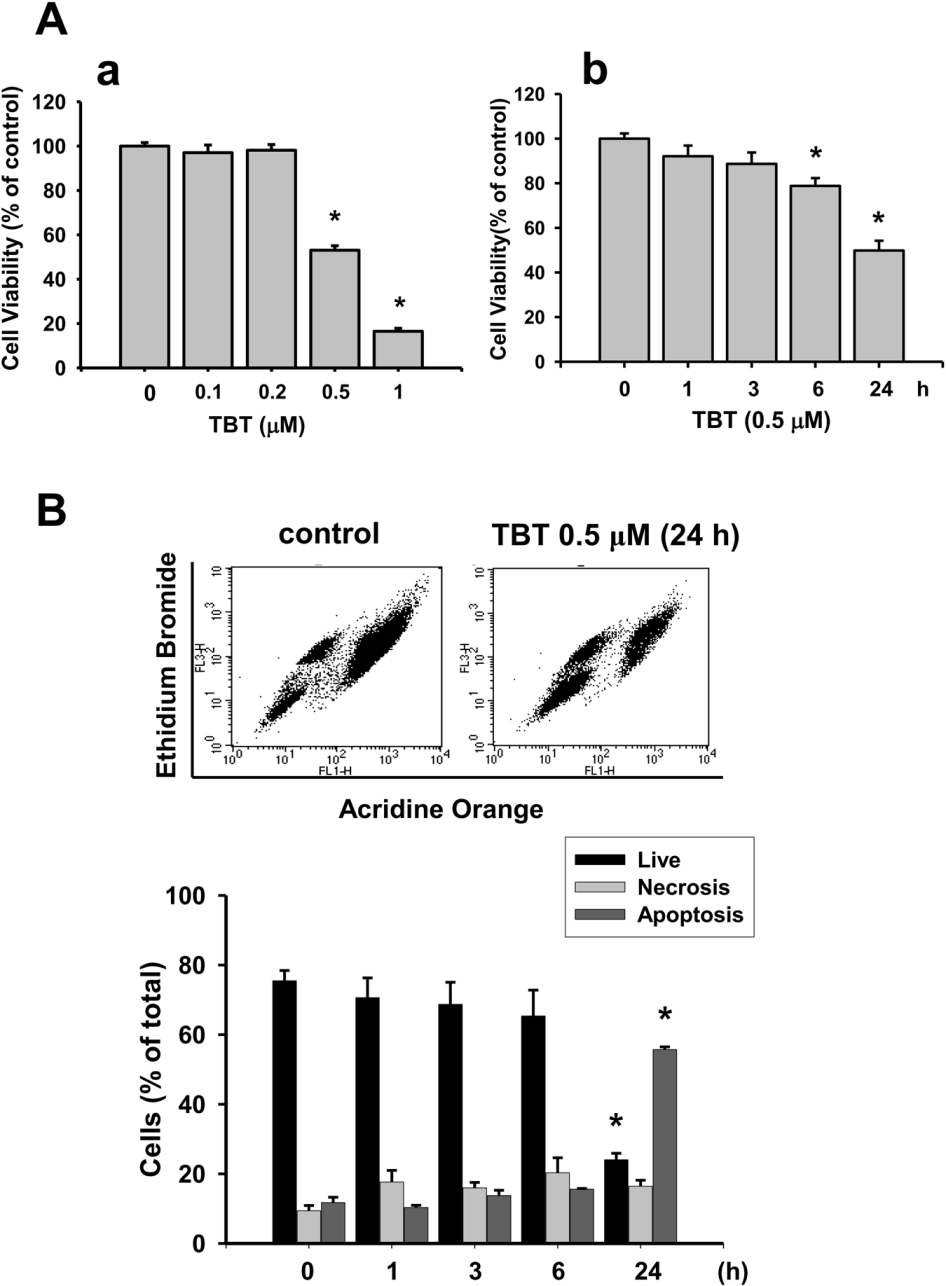
TBT decreased cell viability and induced apoptosis in β-cells. RIN-m5F cells were treated with or without TBT (0.1–1 μM) for 1–24 h. (**A**) Cell viability was determined by MTT assay in a dose-dependent manner (a) and a time-dependent manner (b). (**B**) Apoptotic or necrotic cells were determined by flow cytometry analysis with acridine orange/ethidium bromide fluorescent probes. Data are presented as mean ± SEM of four independent experiments with triplicate determinations in each experiment. *P < 0.05 as compared to vehicle control.

**Figure 2:**
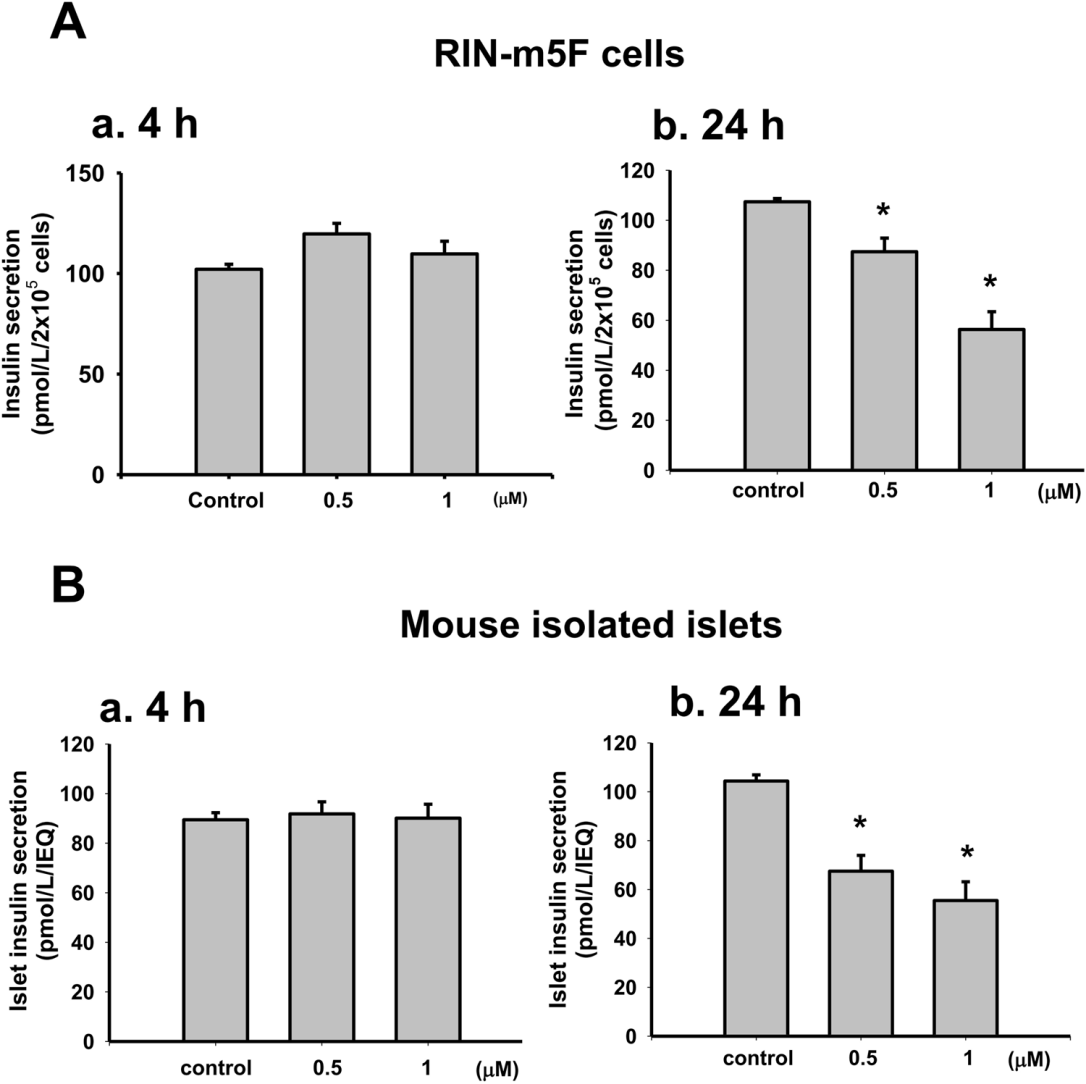
The effects of TBT on insulin secretion in β-cells and islets. Both RIN-m5F cells (**A**) and mouse islets (**B**) were treated with TBT (0.5 and 1 μM) for 4 h (a) or 24 h (b). The glucose (20 mM)-stimulated insulin secretion was detected. The levels of insulin in the solution were determined by ELISA. The secreted insulin was normalized to 2 × 10^5^ cells for RIN-m5F cells or islet equivalent of quality (IEQ) for mouse islets. TBT did not affect the basal insulin secretion at 4 h exposure. Data are presented as mean ± SEM of three independent experiments with triplicate determinations in each experiment. *P < 0.05 as compared to vehicle control.

We first investigated whether TBT induced cytotoxicity in pancreatic β-cells. Treatment with low-concentration TBT (0.1-1 μM) for 24 h significantly reduced the RIN-m5F cell viability in a dose-dependent manner (Fig. 1A-a). The median lethal concentration was approximately 0.5 μM. TBT at 0.5 μM time-dependently reduced the RIN-m5F cell viability that was 78.8 ± 3.5% control at 6 h and 49.8 ± 4.4% control at 24 h (Fig. 1A-b). We further investigated the effects of TBT on β-cell function determined by glucose-stimulated insulin secretion assay. The glucose-stimulated insulin secretion was not affected in RIN-m5F cells (Fig. 2A-a) or isolated mouse islets (Fig. 2B-a) treated with TBT 0.5 and 1 μM for 4 h. TBT at the concentrations of 0.5 and 1 μM significantly decreased the glucose-stimulated insulin secretion in RIN-m5F cells (Fig. 2A-b) and isolated mouse islets (2B-b) after 24 h treatment.

**Figure 3:**
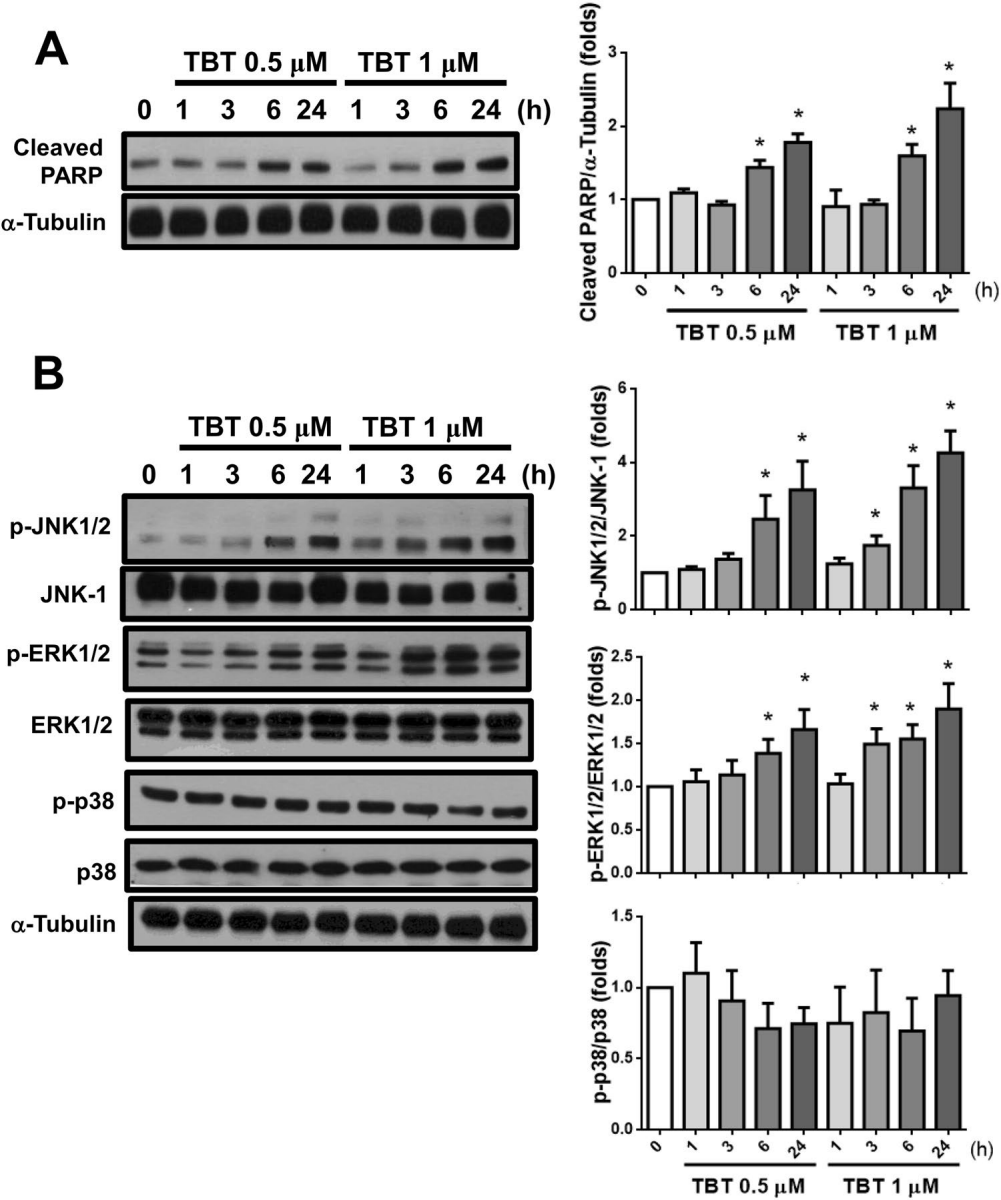
Effects of TBT on the PARP cleavage and phosphorylations of MAPKs in β-cells. RIN-m5F cells were treated with TBT (0.5 and 1 μM) for 1–24 h. The PARP cleavage (**A**) and protein phosphorylations of JNK-, ERK1/2-, and p38-MAPKs (**B**) were analyzed by Western blot. The quantification was determined by densitometric analysis. Data are presented as mean ± SEM of three independent experiments with triplicate determinations in each experiment. *P < 0.05 as compared to vehicle control.

In order to examine whether apoptosis was involved in TBT-induced pancreatic β-cell cytotoxicity, we analyzed the apoptotic cell population using flow cytometry. As shown in Fig. 1B, treatment of RIN-m5F cells with TBT (0.5 μM) for 24 h markedly decreased the number of live cells and significantly increased the number of apoptotic cells. To further assess the apoptotic signaling induced by TBT, the activation of PARP and MAPKs was investigated. As shown in Fig. 3A, exposure of RIN-m5F cells to 0.5 and 1 μM TBT for 6 h resulted in the cleavage of PARP and maintained to 24 h. Moreover, the levels of phosphorylation in MAPKs-related signaling molecules JNK and ERK1/2, but not p38, were markedly increased after treatment of RIN-m5F cells with 0.5 and 1 μM TBT (Fig. 3B).

### TBT-induced apoptosis is mediated by ROS-regulated the activation of JNK pathway in β-cells

**Figure 4:**
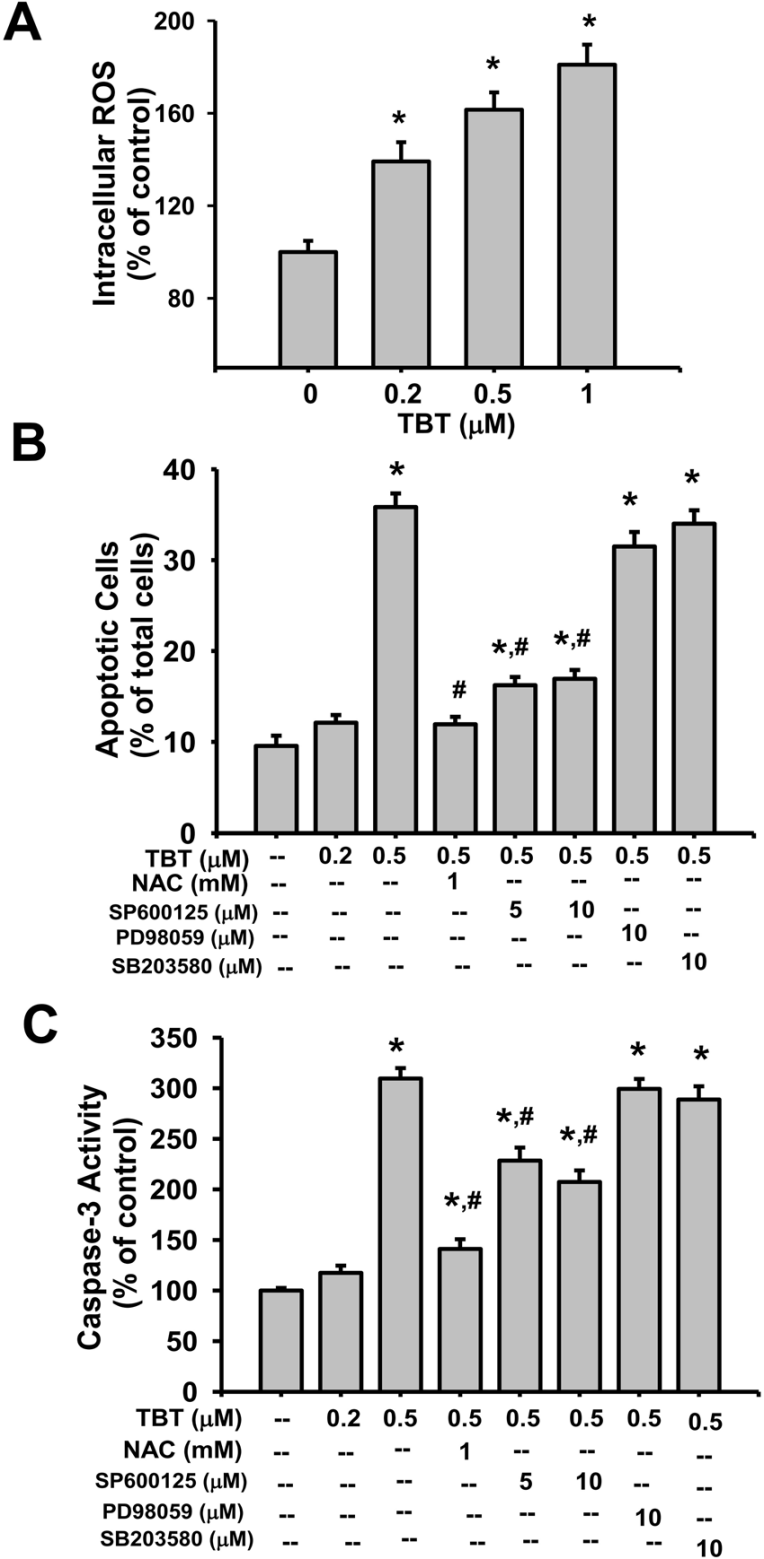
Involvement of ROS and JNK signals in the TBT-induced apoptosis and caspase-3 activity in β-cells. (**A**) RIN-m5F cells were treated with or without TBT (0.2–1 μM) for 1 h. The generation of ROS was measured by flow cytometry with peroxide-sensitive fluorescent probe. (**B**,**C**) RIN-m5F cells were treated with TBT (0.2 and 0.5 μM) for 24 h in the presence or absence of antioxidant N-acetylcysteine (NAC, 1 mM), JNK inhibitor SP600125 (5 and 10 μM), ERK1/2 inhibitor PD98059 (10 μM), or p38-MAPK inhibitor SB203580 (10 μM). Apoptotic cells were determined by flow cytometry analysis with acridine orange/ethidium bromide fluorescent probes (**B**). Caspase-3 activity was detected by CaspACE^TM^ fluorometric activity assay kit (**C**). Data are presented as mean ± SEM of four independent experiments with triplicate determinations in each experiment. *P < 0.05 as compared to vehicle control. ^#^*P* < 0.05 as compared to TBT alone.

We next examined the role of ROS in TBT-induced β-cell injury. As shown in Fig. 4A, RIN-m5F cells treated with TBT (0.2-1 μM) for 1 h markedly increased the intracellular ROS generation in a dose-dependent manner.

**Figure 5:**
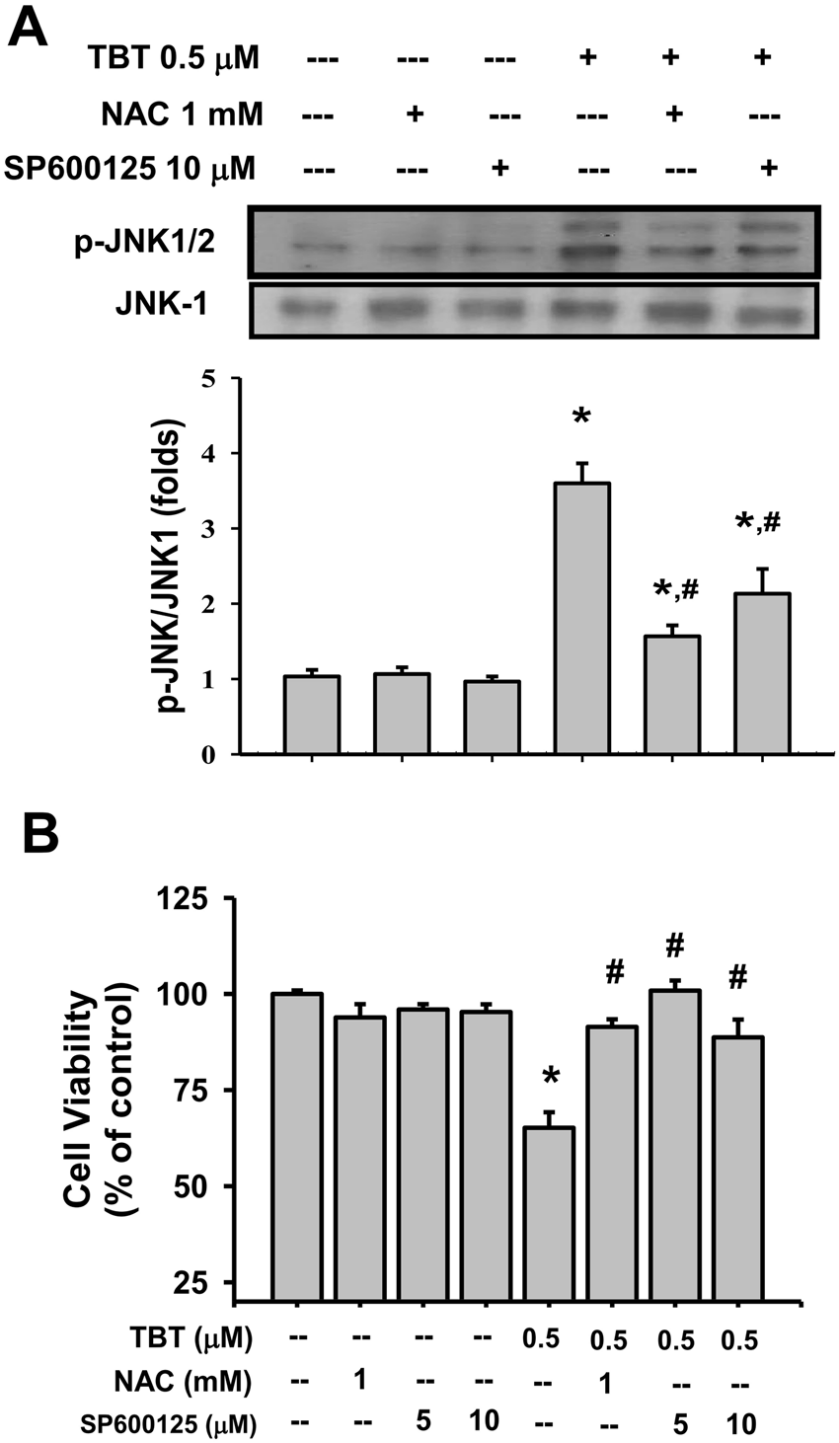
Involvement of ROS signaling in the TBT-induced JNK phosphorylation and decreased cell viability in β-cells. RIN-m5F cells were treated with or without TBT (0.5 μM) for 24 h in the presence or absence of NAC (1 or 2 mM) or SP600125 (5 or 10 μM). (**A**) JNK phosphorylation was determined by Western blot. The quantification was determined by densitometric analysis. (**B**) Cell viability was determined by MTT assay. Data are presented as mean ± SEM of three independent experiments with triplicate determinations. *P < 0.05 as compared to the vehicle control. ^#^*P* < 0.05 as compared to TBT alone.

TBT at 0.5 μM, but not 0.2 μM, significantly increased apoptosis induction (Fig. 4B) and caspase-3 activity in β-cells (Fig. 4C). Pretreatment with antioxidant N-acetylcysteine (NAC) could effectively prevent the increase of apoptosis (Fig. 4B) and caspase-3 activity (Fig. 4C) induced by TBT (0.5 μM) in β-cells. MAPKs-mediated signaling pathways are known to play the roles in toxic insults- or ROS-induced apoptosis^20^. We next elucidated the relationship between ROS and MAPKs activation in TBT-induced β-cell apoptosis. A specific JNK inhibitor (SP600125; 5 and 10 μM), but not an ERK1/2 inhibitor (PD98059; 10 μM) and a p38-MAPK inhibitor (SB203580; 10 μM), significantly reversed the TBT (0.5 μM)-induced β-cell apoptosis (Fig. 4B) and caspase-3 activity (Fig. 4C). Moreover, both NAC and SP600125 effectively reversed the TBT (0.5 μM)-induced JNK phosphorylation (Fig. 5A) and decrease of cell viability (Fig. 5B). These results indicated that ROS may contribute the activation of JNK signaling leading to submicromolar-concentration TBT-induced β-cell apoptosis.

### TBT alters glucose metabolism and increased plasma lipid peroxidation in mice

**Figure 6:**
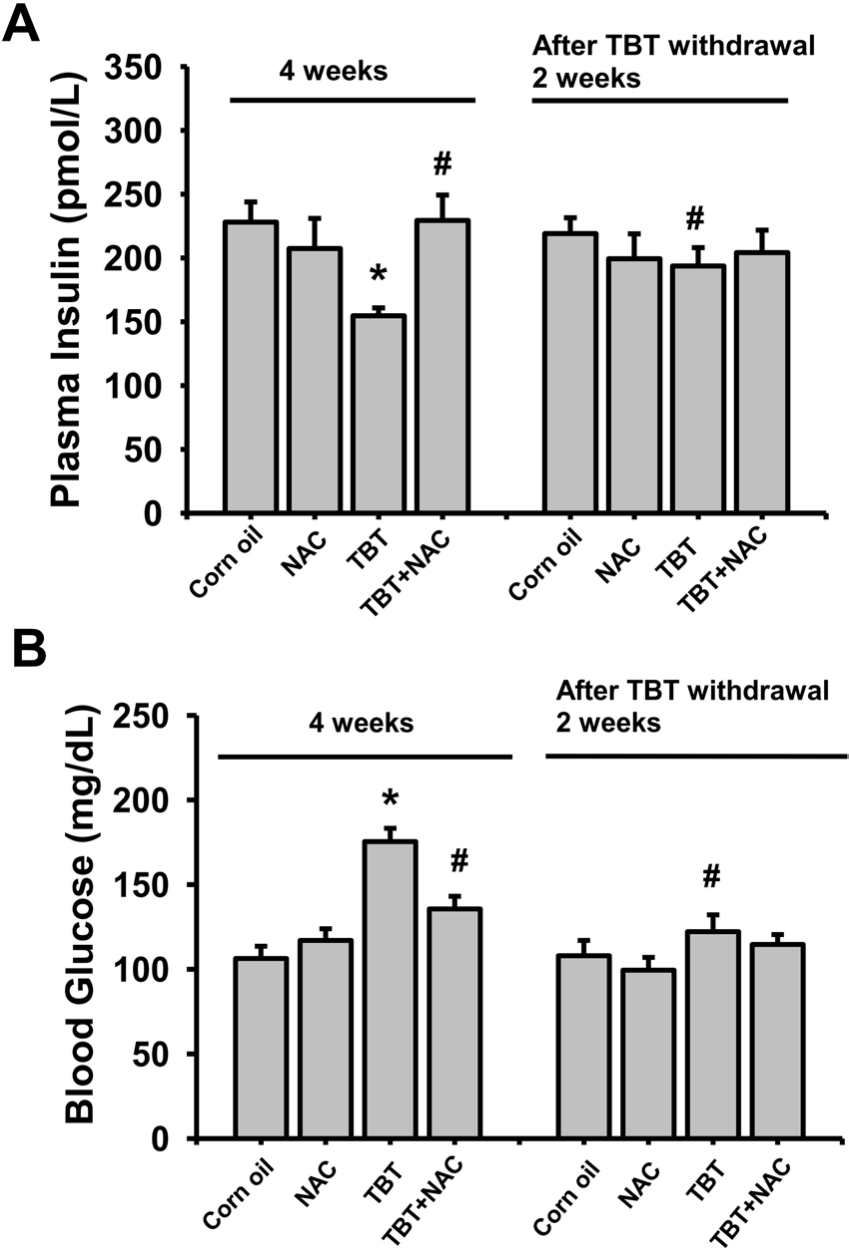
TBT increased blood glucose and decreased plasma insulin in mice. Mice were orally gavaged with TBT (0.25 mg/kg) in the presence or absence of NAC (150 mg/kg) for 4 consecutive weeks. In some experiments, mice were treated with TBT for 4 weeks and then the exposure was terminated for 2 week recovery period. The levels of plasma insulin (**A**) and blood glucose (**B**) were detected. Data are presented as mean ± SEM (n = 10). *P < 0.05 as compared to the vehicle control (corn oil). ^#^*P* < 0.05 as compared to TBT alone.

**Figure 7:**
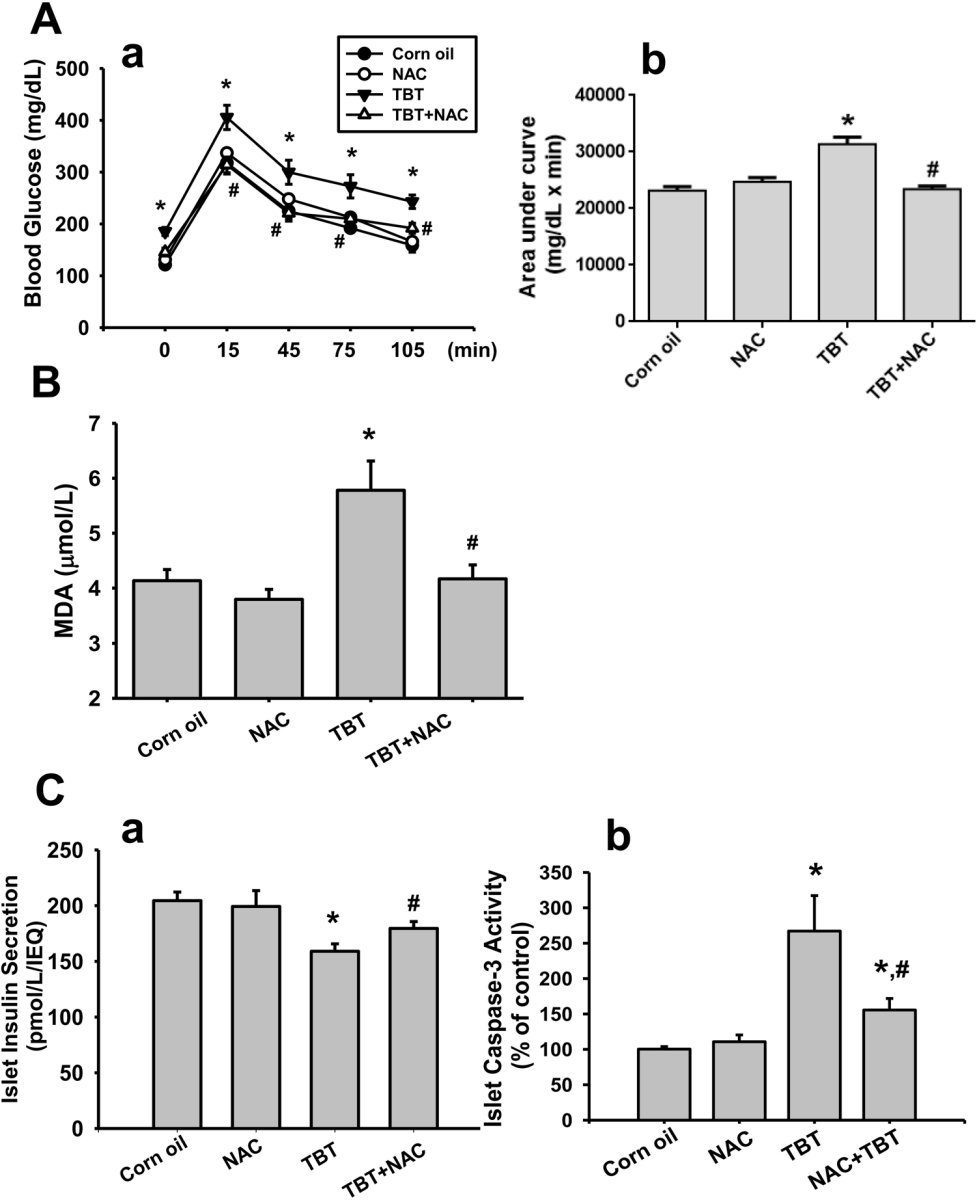
*In vivo* effects of TBT on glucose tolerance, lipid peroxidation, islet insulin secretion, and islet caspase-3 activity. Mice were orally gavaged with TBT (0.25 mg/kg) in the presence or absence of NAC (150 mg/kg) for 4 consecutive weeks. Oral glucose tolerance test was assayed (**A**) and plasma malondialdehyde (MDA) level was detected (**B**). The area under the curve (AUC) for OGTT was calculated (**A**-**b**). Moreover, the islets were isolated from the pancreases of TBT-treated mice. The glucose-stimulated insulin secretion (**C**-**a**) and caspase-3 activity (**C**-**b**) in islets were detected. The levels of insulin in the solution were determined by ELISA. Data are presented as mean ± SEM (n = 10). *P < 0.05 as compared to the vehicle control (corn oil). ^#^*P* < 0.05 as compared to TBT alone.

We next examined the effects of TBT on the changes of blood glucose and insulin levels and islet function in mice. Treatment with TBT (0.25 mg/kg/day) for 4 consecutive weeks showed a significant decrease in plasma insulin levels (Fig. 6A) and a significant increase in blood glucose levels (Fig. 6B). TBT-exposed mice also obviously caused the glucose intolerance that the curve of blood glucose for oral glucose tolerance test (OGTT) (Fig. 7A-a) and area under the curve (AUC) (Fig. 7A-b) were significantly elevated as compared to vehicle control. Moreover, the result of plasma lipid peroxidation assay after exposure of TBT to mice revealed that the plasma malondialdehyde (MDA) levels were dramatically increased (Fig. 7B). We next isolated the islets from the treated mice to show β-cell function impairment and apoptosis induction. As shown in Fig. 7C-a, the glucose-stimulated insulin secretion was significantly decreased in islets isolated from TBT-treated mice. The caspase-3 activity was also significantly increased in islets isolated from TBT-treated mice (Fig. 7C-b). These *in vivo* effects of TBT could be significantly reversed by treatment of NAC (Figs 6 and 7).

For testing the reversibility of TBT-induced *in vivo* toxicity, mice were treated with TBT for 4 weeks and then the exposure was terminated for 2-week recovery period. After removing the TBT exposure for 2 weeks, the decreased plasma insulin levels (Fig. 6A) and elevated blood glucose levels (Fig. 6B) were significantly reversed.

## Discussion

EDCs are discharged into the environment and remain to be easily introduced into organisms and act as hormones, and more than 100 species of EDCs have been identified including TBT^6,7^. TBT is available presence in food and drinking water by contamination with industrial effluents and leaching. A high TBT concentration of 1510 ng per g dry weight in oysters has been found in Hsiangshan coastal area of Taiwan^21^. The concentrations of TBT in muscles of fish obtained from Taiwan harbors were observed ranging from 4.2 to 3389.7 ng per g wet weight^7^. Miki *et al*. have found that the considerable blood TBT levels of marine fish in northern Kyushu coastal area of Japan are 1.4–190 ng/mL^22^. Kannan *et al*. have shown that the total concentrations of organotins in all of the house dust samples from Albany, New York, USA are ranging from 390 to 28,000 ng/g^23^. The blood TBT levels have been detected and up to 85 ng/mL (261 nM) in the volunteers from Michigan (USA)^24^. In the present study, we used the doses of TBT *in vitro* (0.5-1 μM) and *in vivo* (0.25 μg/g) to investigate the TBT-induced pancreatic β-cell injury and dysfunction. The results showed that low-dose TBT is capable of inducing β-cell injury and insulin secretion inhibition. TBT induced the activation of caspase-3 and triggered apoptotic cell death.

Oxidative stress is known to be involved in the pathophysiological processes of pancreatic β-cell death and dysfunction during diabetes development^17,25^. ROS is known to play an important role in TBT-triggered toxicological effects *in vivo* and *in vitro*^10–12,26^. The toxicological effects of TBT (0.01–1 μM) in various types of mammalian cells, including hepatocytes, thymocytes, sertoli-germ cells, and cerebral cortical cells, have been reported^10–12,18^. The involvement of both ROS- and JNK-related signaling pathways has been shown in the TBT (2 μM)-induced PC12 cell apoptosis^26^. Zhang *et al*. have also found that ROS-mediated MAPKs activation is an upstream mechanism for TBT-induced apoptosis in mouse livers^12^. In addition, ROS promoting apoptosis mediated by the activation of MAPKs (JNK, ERK, and p38) has been identified to be an important mechanism for β-cell apoptosis induced by arsenic^27^, high glucose^28^, and free fatty acids^29^. In this study, low-concentration TBT (0.5-1 μM) caused an increase in ROS production, an induction of apoptosis, and a reduction in cell viability in pancreatic β-cells, which could be effectively prevented by treatment of antioxidant NAC. Moreover, treatment with TBT to β-cells significantly increased the levels of JNK and ERK1/2-MAPK. Pretreatment of both NAC and JNK inhibitor SP600125, but not both ERK inhibitor PD98059 and p38 inhibitor SB203580, dramatically abrogated the TBT-induced apoptosis and dysfunction in β-cells. These findings suggest that low-dose TBT can induce an ROS-triggered JNK signaling pathway to activate apoptotic cell death.

Evidence reveals the crucial *in vivo* effects of TBT exposure on the development of pancreatic β-cell injury, leading to glucose homeostasis disruption and insulin secretion dysfunction. The studies have indicated that high-dose triphenyltin exposure can inhibit the insulin secretion and interfere with glucose metabolism in hamster and rabbit treated with 60 mg/kg and 100 mg/kg of triphenyltin, respectively^14,30^. Short-term exposure of TBT (0.025 mg/kg, 3 weeks) has been found to increase blood glucose and plasma insulin levels, and caused the insulin resistance in mice^13^. Treatment of TBT (0.05 mg/kg) for 45 days has been shown to increase the blood glucose levels accompanied with a decrease in islet cell proliferation and an increase in cell apoptosis^16^. Li *et al*. recently further demonstrated that the hyperglycemia in mice exposed to low-dose TBT for 45 days could be reversed after removing the TBT exposure for 60 days^31^. In the present study, we found that a 4-week consecutive exposure to TBT (0.25 mg/kg) in mice caused an increase in fasting blood glucose, a decrease in plasma insulin, and an elevation in glucose intolerance accompanied by a significant increase of plasma lipid peroxidation. We also found that the glucose-stimulated insulin secretion was significantly decreased and the caspase-3 activity was significantly increased in islets isolated from TBT-treated mice. These TBT-induced responses in mice could be effectively prevented by antioxidant NAC. We also found that after removing the TBT exposure for 2 weeks, the decreased plasma insulin levels and elevated blood glucose levels were significantly reversed. Overall, these findings suggest that low-dose TBT possesses the ability to cause β-cell injury and dysfunction *in vivo* via an oxidative stress-regulated pathway, triggering a reversible hypoinsulinemic hyperglycemia.

Organotin compounds exposure (dibutyltin and dioctyltin; 50–150 ppm in the diet for 2 weeks) to rats has been shown to induce severe thymus atrophy that is reversible after the exposure was terminated for 8 weeks^32^. It has also been found that oral administration of a single dose tributyltin oxide (100 mg/kg) to rats causes adrenal hypertrophy and thymus atrophy, which could be completely recovered within 24 days^33^. In an accidental exposure to trimethyltin vapor (about 3 month exposure), the neuropathic symptoms in patients could be progressively subsided after the exposure was removed for one month^34^. Li *et al*. have recently found that TBT (50 μg/kg for 45 days)-induced hyperglycemia and suppressed insulin receptor signal pathway can be recovered after removing the TBT exposure for 60 days^31^. The present study also found that TBT (0.25 mg/kg for 4 weeks)-induced hypoinsulinemic hyperglycemia could be recovered after the treatment was terminated for 2 weeks. The recovery of toxicities induced by TBT or other organotins after exposure termination under various conditions may be related to the rapid metabolism of organotins. An experimental study of tributyltin exposure (40 mg/kg, oral administration) to rats showed a transient increase in hepatic tributyltin level within 24 h followed by a rapid de-alkylation to decrease the tributyltin content and increase the levels of dibutyltin, monobutyltin, and inorganic tin in the livers^35^. However, the mechanisms of reversibility for TBT-induced toxicities still remain unclear. The regarding cell regeneration of mesenchymal stem cells in pancreatic islets for induction of β-cell differentiation may also be a possible mechanism for the recovery of TBT-induced β-cell toxicity in mice after removal of TBT exposure. The further investigation for mechanisms of reversibility of TBT-induced β-cell toxicity needs to be clarified in the future.

**Figure 8:**
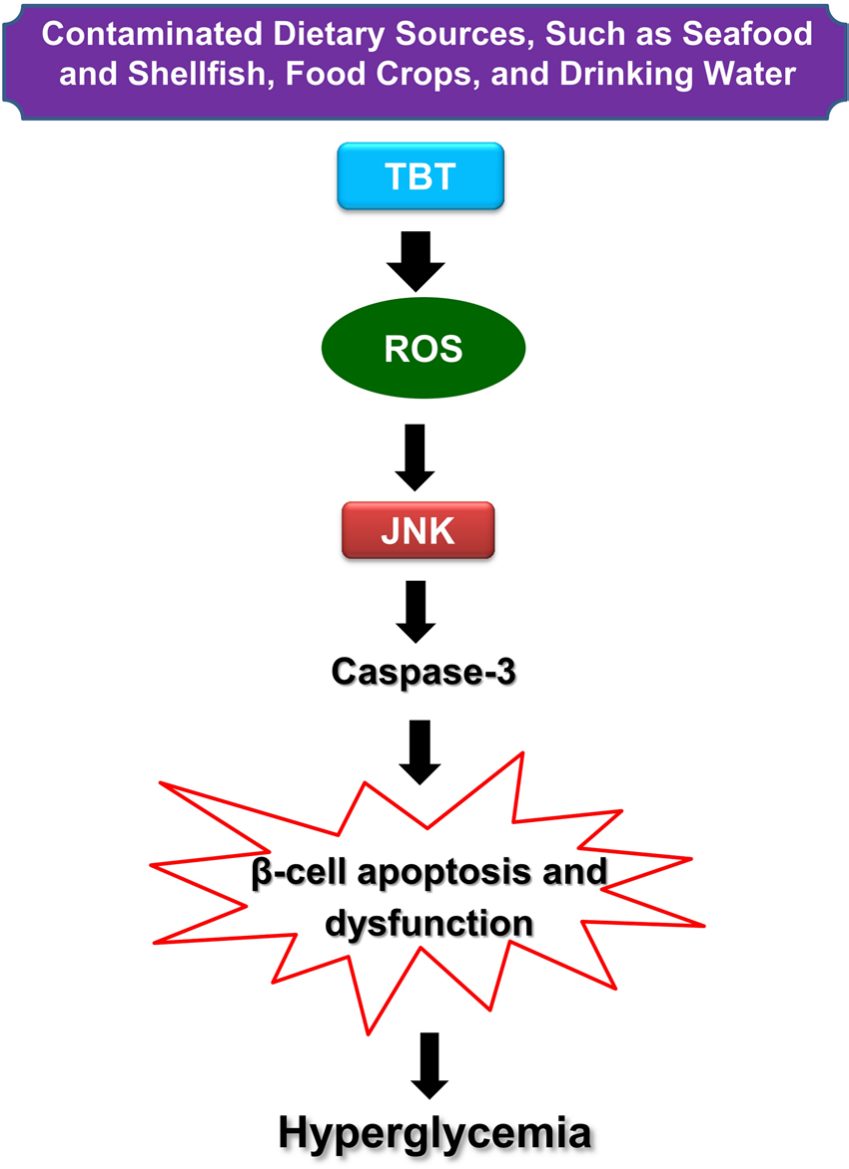
Schematic diagram of the signaling pathways involved in TBT-induced pancreatic β-cell apoptosis and dysfunction.

In conclusion, this study demonstrates that low-dose TBT is capable of inducing β-cell apoptosis and dysfunction. TBT-triggered β-cell apoptosis is caused by activating the JNK pathway. ROS acts as an upstream key signaling molecule in TBT-induced JNK activation. A schematic diagram of the signaling pathways involved in TBT-induced pancreatic β-cell apoptosis and dysfunction was shown in Fig. 8. These findings provide the significant evidence to confirm that TBT is an importantly environmental risk factor for diabetes.

## Materials and Methods

### Cell culture

RIN-m5F cell, a rat pancreatic β-cell line (ATCC, CRL-11605) was used. The completed culture medium was prepared with RPMI 1640 medium, 10% fetal bovine serum (FBS), penicillin (100 U/mL), and streptomycin (100 μg/mL) (Thermo Fisher Scientific, Waltham, MA, USA). RIN-m5F cells were maintained in a standard humidified incubator with a 5% CO_2_ condition at 37 °C. The cells were sub-cultured when reaching 80% confluency.

### Measurement of cell viability

The viability of β-cells was assessed by the MTT assay as described previously^36^. RIN-m5F cells were gentle and average seeded in the 96-well plates (2 × 10^4^ per well) over night. Cells were treated with TBT (Sigma-Aldrich, St. Louis, MO, USA) at the concentrations of 0.1–1 μM for 1–24 h, and then changed to the fresh medium contained 3-(4,5-dimethyl thiazol-2-yl-)−2,5-diphenyl tetrazolium bromide (MTT; 0.2 mg/mL). After incubation for 4 h, cells were treated with dimethyl sulfoxide (DMSO) solution. The detection for absorbance at 570 nm was performed by a microplate reader.

### Measurement of glucose-stimulated insulin secretion

The glucose-stimulated insulin secretion assay in RIN-m5F cells or islets was performed as described previously^37^. Briefly, the cultured cells or islets were moved to the solution with Krebs Ringer buffer contained 5.5 mM glucose and then transferred to the solution with 20 mM glucose for a 1-h incubation in a 5% CO_2_ incubator at 37 °C. An insulin antiserum immunoassay kit purchased from Mercodia (Uppsala, Sweden) was used to measure insulin levels by a manufacturer’s protocol. The levels of insulin in the solution were determined by ELISA. The detection for absorbance at 450 nm was performed by an ELISA reader. The secreted insulin was normalized to 2 × 10^5^ cells for RIN-m5F cells or islet equivalent of quality (IEQ) for mouse islets.

### Determination of ROS production

Cells were seeded at 2 × 10^5^ cells/well in a 24-well plate. Cells were exposed to TBT at the concentrations of 0.2 to 1 μM for 1 h. Subsequently, cells were changed to the fresh medium contained with 2′,7′-dichlorofluorescin diacetate (DCFH-DA; 20 μM) (Molecular Probes, Eugene, OR, USA) for 15 min at 37 °C. The intracellular ROS levels were detected by a flow cytometer (FACScalibur, Becton Dickinson, Sunnyvale, CA, USA). Moreover, in the experiment for detection of islet ROS, about 100 islets isolated from five separate isolations of mouse pancreases were used for each group.

### Determination of apoptosis using the acridine orange/ethidium bromide assay

The acridine orange/ethidium bromide assay was performed as previously described^38^. Briefly, RIN-m5F cells were exposed to TBT (0.5 μM) for 24 h, and then total floating and adherent cells were collected with centrifuge (160 × g, 5 min). The condense cells were re-suspended by the PBS and then cells were incubated with the acridine orange and ethidium bromide for 5–10 min. The fluorescence was analyzed using a flow cytometer (FACScalibur, Becton Dickinson). Live, apoptotic, and necrotic populations were analyzed using Win-MDITM flow analysis software.

### Measurement of caspase-3 activity

The caspase-3 activity assay in RIN-m5F cells or mouse islets was performed as described previously^39^. RIN-m5F cells were exposed to TBT (0.5 μM) with or without the treatments of NAC, PD98058, SB203580, or SP600125, which were added 1 h prior to the treatment with TBT, at 37 °C. Cell lysates were incubated at 37 °C with 10 μM Ac-DEVD-AMC, a caspase-3/CPP32 substrate (Promega Corporation, Madison, WI, USA), for 1 h. The fluorescence was determined using a spectrofluorometer. The protein levels of cell lysates were determined with the bicinchoninic acid protein assay kit (Pierce, Rockford, IL, USA) to normalize the cell numbers between the control and TBT-treated groups.

### Western blotting analysis

The protein expressions were performed by Western blotting as previously described^37^. Briefly, the protein samples (50 μg) of each cell lysate were subjected to electrophoresis on 10% SDS-polyacrylamide gels. The protein samples were electroblotted on polyvinylidene difluoride membranes and then blocking. The blots were incubated with antibodies for cleaved poly (ADP-ribose) polymerase (PARP) (Cell Signaling Technology, Danvers, MA, USA), phosphorylated extracellular signal-regulated kinase (ERK)1/2, ERK1/2, phosphorylated Jun N-terminal kinase (JNK), JNK, phosphorylated p38 mitogen-activated protein kinase (MAPK), p38, and α-tubulin (Santa Cruz Biotechnology, Santa Cruz, CA, USA). The membranes were then incubated with horseradish peroxidase-conjugated secondary antibodies. An enhanced chemiluminescence reagent (BioRad Laboratories, Redmond, WA, USA) was used to depict the protein bands on membranes. The gel band quantitative densitometric analysis was determined by the image J 1.48 software (National Institutes of Health, Bethesda, MD, USA).

### Animals

The male CD-1 mice (20–25 g) were purchased from the Animal Center of the College of Medicine, National Taiwan University. All animal studies were approved by the ethical review committee of College of Medicine, National Taiwan University, and followed to the regulations of Taiwan and NIH (USA) guidelines on the care and welfare of laboratory animals. Mice were humanly housed in a room with temperature of 22 ± 2 °C and 12-h light/dark cycles. Animals were randomly divided into four groups [control (corn oil), TBT (0.25 mg/kg), TBT + NAC (150 mg/kg), and NAC alone]. Each group contained 10 mice. The experimental period was 4 weeks. In some experiments, mice were treated with TBT for 4 weeks and then the exposure was terminated for 2 week recovery period. Mice were sacrificed with anesthesia (intraperitoneal injection of pentobarbital 50 mg/kg) at the end of experiments.

### Measurements of glucose and insulin in blood samples

Mouse tail vein bloods were sampled and determined the blood glucose by a glucose analyzer (Horiba Industry, Kyoto, Japan). The measurement of plasma insulin was performed by an immunoassay kit (Mercodia, Uppsala, Sweden).

### Oral glucose tolerance test (OGTT)

The experimental procedure was followed to our previous study^40^. Briefly, after fasting, control and TBT-treated mice were orally challenged with glucose (1 g/kg). The tail vein bloods were sampled at adaptive time points (before and after glucose challenge at 15, 45, 75, and 105 min). The area under the curve (AUC) for OGTT was calculated using GraphPad Prism 6.

### Lipid peroxidation assay

The plasma samples were collected and the malondiadehyde (MDA) levels were assayed immediately using a lipid peroxidation assay kit (Calbiochem, Burlington, MA, USA). The absorbance at 586 nm was detected by an ELISA micro-plate reader.

#### Mouse islet isolation

The method of islet isolation from the mouse pancreases using collagenase digestion was performed as described previously^37^. Briefly, islets were handpicked after enzyme digestion and Ficoll gradient separation, and then cultured in RPMI-1640 medium supplemented with 10% FBS and penicillin (100 U/mL) and streptomycin (100 μg/mL) in a 5% CO_2_ incubator at 37 °C. Islets were counted using dithizone staining, and calculated the islet equivalent of quality (IEQ; 1 IEQ = 150 μm)). The islets with 75–150 μm in diameter were used.

### Statistical analysis

All data are expressed as mean ± standard errors of the means (SEM). Multiple comparisons among different groups were analyzed using one-way analysis of variance followed by Holm-Sidak post hoc test. A *P* value less than 0.05 was considered statistically significant. A software of SPSS-16.0 was used for statistical analysis.
